# Structural Damage Diagnosis-Oriented Impulse Response Function Estimation under Seismic Excitations

**DOI:** 10.3390/s19245413

**Published:** 2019-12-09

**Authors:** Jian-Fu Lin, Junfang Wang, Li-Xin Wang, Siu-seong Law

**Affiliations:** 1 Center of Safety Monitoring of Engineering Structures, Shenzhen Academy of Disaster Prevention and Reduction, China Earthquake Administration, Shenzhen 518003, China; linjianf@hotmail.com (J.-F.L.); wlx@szadpr.cn (L.-X.W.); 2 College of Civil and Transportation Engineering, Shenzhen University, Shenzhen 518060, China; 3 School of Civil Engineering, Chongqing University, Chongqing 400045, China; siu-seong.law@connect.polyu.hk

**Keywords:** impulse response function estimation, multiple excitations, structural damage diagnosis, inverse problem

## Abstract

Impulse response function (IRF) is an ideal structural damage index for the identification of structural damage associated with changes in modal properties. However, IRFs from multiple excitations applied at different degrees-of-freedoms jointly contribute to the dynamic response, and their estimation is often underdetermined. Although some efforts have been devoted to the estimation of IRF for a structure under single excitation, the case under multiple excitations has not been fully investigated yet. The estimation of IRF under multiple excitations is generally an ill-conditioned inverse problem such that an incorrect or non-feasible solution is common, preventing its application to damage detection. This work explores this problem by introducing dimensionality reduction transformation matrices relating two sets of IRFs of a structure with discussions on the performance of the non-unique transformation matrices. Then, the extraction of IRF via wavelet-based and Tikhonov regularization-based methods are compared. Finally, a numerical study with a truss structure is conducted to validate the estimation of the IRFs and to demonstrate their applicability for damage detection under seismic excitations. Both the damage locations and severity are accurately identified, indicating the proposed methodology can enable the IRFs estimation under multiple excitations for successful damage detection.

## 1. Introduction

**Figure 1:**
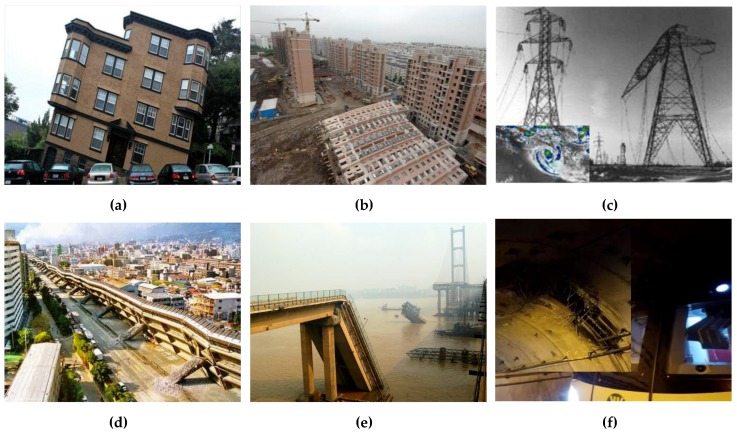
Typical damages for different kinds of civil structures: (**a**) Soil settlement-induced damage; (**b**) construction work-induced collapse; (**c**) strong wind-induced buckling; (**d**) severe earthquake-induced collapse; (**e**) ship collision-induced damage; (**f**) foreign object (pile) invasion-induced damage.

Structural health monitoring (SHM) has played an important role in the fields of mechanical and civil engineering in the last few decades. Early detection of damage in a structure during its service life has attracted much attention because structural degradation is inevitable once structures are built. Many civil structures are currently suffering from local damages. If these local damages cannot be identified in time, serious global structural failures may happen from extreme loadings or accidents, as shown in Figure 1. The occurrence of damage in a structure due to the change of physical properties produces changes in a structure’s dynamic characteristics, such as its natural frequencies, mode shapes, modal damping, frequency response function (FRF), impulse response function (IRF), etc. The understanding and application of these changes are needed for the identification of damage locations and severities. Comprehensive reviews [1,2,3] on vibration-based damage detection methods have been performed. Natural frequencies, mode shapes, and their derivatives (e.g., model shape curvature, flexibility matrixes, and modal strain energy, etc.) are usually taken as damage detection indices in the frequency domain. However, natural frequencies have been found insufficiently sensitive to detect local damage in a highway bridge structure [4], and measurement errors in mode shapes extraction often result in large false alarms in mode shape-based damage detection [5]. Nagarajaiah and Yang et al. [6,7,8] have devoted to techniques in improving the accuracy of output-only modal identification for structural health monitoring. Recently, Zhou et al. proposed a transmissibility-based damage detection method, which is feasible for identifying damage in pipelines and bridges [9,10].

Since damage detection indices in frequency-domain are sometimes not sufficiently sensitive to local damages when a limited number of sensors are available, other researchers directly used structural dynamic responses for model updating and damage detection without having modal extractions. Cattarius and Inman [11] employed phase shift in the time history of structural vibration response to identify the presence of local anomalies in a plate-like structure as well as a helicopter blade section. Choi and Stubbs [12] formed the damage index directly from the time response to locate and quantify local anomalies in a structure. Kang et al. [13] presented a system identification scheme in time-domain to estimate stiffness and damping parameters of a structure using measured accelerations, and a regularization technique was employed to alleviate the ill-condition of system identification. Later, Lu and Law [14] developed a response sensitivity-based damage detection method to solve the structural damage detection problem in time-domain, which could identify local changes in structure from a few measurement locations. However, the updated results were subject to the effect of measurement noise. More recently, Lin and Xu [15,16,17,18,19] proposed a covariance-based multi-sensing damage detection method with optimal sensor placement in which the damage index was sensitive to local damage but insensitive to measurement noise. Other researchers also developed data-driven damage detection methods incorporating artificial intelligence algorithms [20,21,22,23,24,25,26].

Most of the above time-domain damage detection approaches rely on the need of an externally applied excitation. It is noted that most of the existing health monitoring systems for civil structures operate continuously, and only ambient excitation can be used for the condition evaluation of these structures. Moreover, structural health monitoring based on ambient excitation (e.g., environmental excitation or traffic vibration) would be the most desirable approach because of its low cost and easy operation. Li and Law [27] have proposed a structural condition assessment approach under ambient white noise excitation. This has been further developed by Li and Law [28] and Law et al. [29], where a new covariance of covariance (CoC) matrix was formed from autocorrelation/cross-correlation functions of structural-acceleration responses. The components of the CoC matrix were found more sensitive to local stiffness reduction than modal frequencies and mode shapes. Researchers [30,31] suggested conducting structural safety assessments by using ambient vibration data from different seismic stations throughout regions that are prone to earthquakes. They took advantage of the microtremor of the earth, which could provide sufficient energy for damage detection. Ma et al. [32] presented a structural damage diagnosis and assessment method for detecting, locating, and quantifying structural damage by directly using structural vibration under seismic excitation. Limongelli [33] proposed an interpolation damage detection method for frames under seismic excitation.

The above time-domain damage detection methods often rely on the damage-induced change of structural dynamic responses, such as acceleration, displacement, strain or stress response, before and after damage occurrence. Dynamic responses are, however, subjected to the variation of excitations. On the other hand, IRFs are intrinsic functions of the system, which are related only to excitation location. To reduce uncertainties in the excitation, IRF, instead of the corresponding dynamic response, is considered as a suitable candidate in the damage detection process. Robertson et al. [34,35] have addressed the use of a discrete wavelet transform (DWT) for the estimation of IRFs from the vibration records. Their research also has shown that the discrete wavelet transformation-based method outperforms the Fourier transformation-based method in extracting the IRFs. Law and Li [36] proposed the IRFs as a damage index for damage detection, and the IRFs were estimated via a discrete wavelet transform approach from acceleration responses of a structure under a sinusoidal force. Li and Law [37] extended the IRFs extraction from acceleration responses of a structure under support excitation for damage identification, avoiding the limitation of requiring a large amount of input energy. Later, Li and Wang [38] developed a new covariance matrix formulated from the IRF of acceleration responses for damage detection with satisfactory results. Li and Hao et al. [39] integrated IRF extraction with optimal sensor placement for damage detection. Recently, Li et al. [40] proposed a damage identification method with a fusion of estimates from the covariance of IRF in different frequency bands. All the above literatures on the extraction of IRF for damage detection consider only single excitation of a structure. Few efforts have been devoted to the estimation of IRF function for a structure under multiple excitations. For example, Law and Lin [41] proposed the extraction of IRF via Tikhonov regularization from acceleration responses of a structure under multiple excitations. However, the estimation of IRF is usually an ill-conditioned inverse problem such that an incorrect or non-feasible solution is common, preventing its application in damage detection, especially for a structure under multiple excitations.

Since the IRFs with multiple excitations applied to different degrees-of-freedom of the structure jointly contribute to a dynamic response, their estimation is most probably underdetermined. Moreover, the dimensionality reduction transformation matrices and IRFs extraction methods are not unique. To explore this further, the authors in [41] and their colleagues will discuss IRF estimation by using another dimensionality reduction transformation matrix, as well as compare the IRF estimation from two extraction techniques in this paper. The dimensionality reduction transformation matrices relating two sets of IRFs of a structure is firstly introduced. A better matrix is then obtained after evaluating the performance of the non-unique transformation matrices. Secondly, the extraction of the IRF via wavelet-based and Tikhonov regularization-based extraction methods are described and compared. Thirdly, an optimal sensor placement method based on the principle of minimizing the estimation error is further proposed in this paper. Finally, numerical study with a plane truss structure is conducted to verify the estimation of the IRFs and demonstrate its application in damage detection. The damage detection results indicate that the regularization-based approach has a more superior transformation matrix, giving an accurate and satisfactory inverse solution. Its application to structural damage detection is also robust to measurement noise.

## 2. Methodology

### 2.1. The Impulse Response Function

The equation of motion of a linear damped structural system with N degrees-of-freedom (DOFs) can be written as follows when under a unit impulse excitation:

(1) $$M\ddot{x}(t)+C\dot{x}(t)+Kx(t)=D\cdot \delta (t)$$

where δ(t) is the Dirac delta function. 

The impulse response function (IRF) is the response function of a system under the input of a unit pulse and is an intrinsic function of the structural system. When the system is in static equilibrium initially, the IRF can be computed from the following using the Newmark method as

(2) $$\left\{ \begin{array}{l} M\ddot{h}(t)+C\dot{h}(t)+Kh(t)=0\\ h(0)=0,\dot{h}(0)=M^{-1}D \end{array}\right.$$

where h(t), $\dot{h}(t)$, and $\ddot{h}(t)$ are the N×1 IRF of displacement, velocity, and acceleration vectors, respectively.

The following studies mainly consider IRF estimation from acceleration responses due to its relative ease of measurement and high signal-to-noise ratio. The estimation of IRFs from displacement or velocity responses is similar. When the structural system is under a single excitation f(t), the acceleration response ${\ddot{x}}_{l}(t)$ from location l at time t can be obtained from the Duhamel integral as:

(3) $${\ddot{x}}_{l}(t)=\int_{0}^{t}f(t-\tau ){\ddot{h}}_{l}(\tau )d\tau$$

and when written in discretized form,

(4) $${\ddot{x}}_{l}(p)=\sum_{i=0}^{p}f(p-i){\ddot{h}}_{l}(i)$$

where p is the number of data points. Equation (4) can be rewritten in matrix form as:

(5) $${\ddot{x}}_{l}=F\cdot {\ddot{h}}_{l}$$

with

(6) $${\ddot{x}}_{l}={\left[\begin{array}{l} {\ddot{x}}_{l}(0)\\ {\ddot{x}}_{l}(1)\\ \cdots \\ {\ddot{x}}_{l}(p)\\ \cdots \\ {\ddot{x}}_{l}(n-1) \end{array}\right]}_{n\times 1};\;F={\left[\begin{array}{cccc} f(0) & 0 & \cdots & 0\\ f(1) & f(0) & \cdots & 0\\ \vdots & \vdots & \ddots & \vdots \\ f(n-1) & f(n-2) & \cdots & f(0) \end{array}\right]}_{n\times n};{\ddot{h}}_{l}={\left[\begin{array}{c} {\ddot{h}}_{l}(0)\\ {\ddot{h}}_{l}(1)\\ \begin{array}{l} \cdots \\ {\ddot{h}}_{l}(i)\\ \cdots \end{array}\\ {\ddot{h}}_{l}(n-1) \end{array}\right]}_{n\times 1}$$

It is noted that the excitation f(t) has been implicitly expressed in matrix F and n is the total number of time instants.

The acceleration response ${\ddot{x}}_{l}(t)$ in Equation (3) can also be obtained as

(7) $${\ddot{x}}_{l}(t)=\int_{0}^{t}{\ddot{h}}_{l}(t-\tau )f(\tau )d\tau$$

with a discretized form as

(8) $${\ddot{x}}_{l}(p)=\sum_{i=0}^{p}{\ddot{h}}_{l}(p-i)f(i)$$

When written in matrix form, we have

(9) $${\ddot{x}}_{l}=H_{l}\cdot f$$

with

(10) $$H_{l}={\left[\begin{array}{cccc} {\ddot{h}}_{l}(0) & 0 & \cdots & 0\\ {\ddot{h}}_{l}(1) & {\ddot{h}}_{l}(0) & \cdots & 0\\ \vdots & \vdots & \ddots & \vdots \\ {\ddot{h}}_{l}(n-1) & {\ddot{h}}_{l}(n-2) & \cdots & {\ddot{h}}_{l}(0) \end{array}\right]}_{n\times n};\;f={\left[\begin{array}{c} f(0)\\ f(1)\\ \begin{array}{l} \cdots \\ f(i)\\ \cdots \end{array}\\ f(n-1) \end{array}\right]}_{n\times 1}$$

It is noted that the IRF ${\ddot{h}}_{l}(t)$ is implicitly expressed in matrix $H_{l}$ in Equation (10).

### 2.2. Estimation of IRF

The IRF cannot be obtained directly from the measurement. It has been reported [35,36] that the IRF can be extracted via the wavelet transform from known measured responses and input excitation information to avoid errors in the Fourier transformation process of both the input and output signal. However, the estimation of IRF is generally an ill-conditioned inverse problem when an incorrect or non-feasible solution is not properly constrained. A Tikhonov regularization-based method for IRF estimation from acceleration responses of a structure under multiple excitations has been proposed [41]. Different estimation methods often have different errors in the IRF estimation. This paper will compare the performance of the wavelet-based method and Tikhonov regularization-based method in the estimation of the IRFs. 

#### 2.2.1. IRF Estimation via Discrete Wavelet Transform

The wavelet-based method for estimating the IRF function will be introduced briefly below. Applying the discrete wavelet transform (DWT) to f(t−τ) and ${\ddot{h}}_{l}(\tau )$ in Equation (3) separately, we can get:

(11) $$\begin{array}{c} {\ddot{x}}_{l}(t)=\int_{0}^{t}\left[f_{0}^{DWT}(t)\varphi (\tau )+f_{1}^{DWT}(t)\psi (\tau )+\cdots +f_{2^{j}+k}^{DWT}(t)\psi (2^{j}\tau -k)\right]\\ \times \left[{\ddot{h}}_{l,0}^{DWT}\varphi (\tau )+{\ddot{h}}_{l,1}^{DWT}\psi (\tau )+\cdots +{\ddot{h}}_{l,2^{j}+k}^{DWT}\psi (2^{j}\tau -k)\right]d\tau \end{array}$$

where φ(τ) and ψ(τ) are the scaling function and the mother wavelet function, respectively. Equation (11) can be rewritten in matrix form for the time series with the orthogonal conditions for both the translation and scale of the Daubechies wavelets as:

(12) $${\ddot{x}}_{l}=F^{DWT}\cdot {\ddot{h}}_{l}{}^{DWT}$$

where matrix $F^{DWT}$ and vector ${\ddot{h}}_{l}{}^{DWT}$ are the force matrix F and unit impulse response vector ${\ddot{h}}_{l}$ after discrete wavelet transform, respectively. The vector ${\ddot{h}}_{l}{}^{DWT}$ can be computed from:

(13) $${\ddot{h}}_{l}{}^{DWT}=F^{DWT^{†}}\cdot {\ddot{x}}_{l}$$

and the estimated ${\ddot{h}}_{l}$ is obtained by inverse discrete wavelet transform as:

(14) $${\ddot{h}}_{l}=\mathrm{IDWT}({\ddot{h}}_{l}{}^{DWT})$$

where DWT and IDWT denote the discrete wavelet transform and the inverse discrete wavelet transform operations.

#### 2.2.2. IRF Estimation via Tikhonov Regularization

The Tikhonov regularization method [42] is one of the widely used techniques to solve the ill-conditioned equation effectively. Accordingly, the IRF estimation via Tikhonov regularization can be expressed as

(15) $$\arg\;\underset{{\ddot{h}}_{l}}{\min}f_{obj}({\ddot{h}}_{l})=||F\cdot {\ddot{h}}_{l}-{\ddot{x}}_{l}|{|}_{2}^{2}+\lambda^{2}||{\ddot{h}}_{l}|{|}_{2}^{2}$$

The regularization parameter λ≥0 controls the extent of contribution of the errors to the cost function, and the term ${\ddot{h}}_{l}$ is obtained by minimizing the cost function. An explicit solution can be given as

(16) $${\ddot{h}}_{l}={\left(FF^{T}+\lambda I\right)}^{-1}F^{T}{\ddot{x}}_{l}$$

where I is the identity matrix. Then, the singular value decomposition of F is given as

(17) $$F=U\Sigma V^{T}=\left[u_{1},u_{2},\cdots ,u_{m}\right]\mathrm{diag}\left(\sigma_{1},\sigma_{2},\cdots ,\sigma_{r}\right)\left[v_{1}^{T},v_{2}^{T},\cdots ,v_{n}^{T}\right]$$

Thus, the regularized solution of the estimated ${\ddot{h}}_{l}$ is given by [42]

(18) $${\ddot{h}}_{l}=V{\left(\Sigma^{2}+\lambda I\right)}^{-1}\Sigma U^{T}{\ddot{x}}_{l}=\sum_{k=1}^{r}\frac{\sigma_{k}^{2}}{\sigma_{k}^{2}+\lambda }\frac{u_{k}^{T}{\ddot{x}}_{l}}{\sigma_{k}^{}}v_{k}$$

It is noted that the $\frac{\sigma_{k}^{2}}{\sigma_{k}^{2}+\lambda }$ can be viewed as a filtering factor, which suppresses the solution terms corresponding to the small singular values and make the solution insensitive to disturbances. 

### 2.3. Dimensionality Reduction Transformation

The IRF estimation for a structure under multiple excitations was seldom studied as compared to that when under single excitation [34,35,38,39]. Dimensionality reduction transformation matrices have been suggested [41] to facilitate the IRFs extraction. It is, however, found that the transformation may take up different forms. This paper aims to discuss the performances of two forms of this transformation for structural damage detection and assess the accuracy of different approaches for the IRF estimation. The IRF functions obtained from different excitations will be compared in the following sections.

An engineering structure is, in general, under multiple excitations, and its acceleration response can be expressed explicitly as a linear superposition of the IRFs as:

(19) $$\begin{array}{c} {\ddot{x}}_{l}=F_{e_{1}}\cdot {\ddot{h}}_{l,e_{1}}+F_{e_{2}}\cdot {\ddot{h}}_{l,e_{2}}+\cdots +F_{e_{i}}\cdot {\ddot{h}}_{l,e_{i}}+\cdots +F_{e_{r}}\cdot {\ddot{h}}_{l,e_{r}}+\cdots +F_{e_{m}}\cdot {\ddot{h}}_{l,e_{m}}\\ \;=[\begin{array}{cccccc} F_{e_{1}} & F_{e_{2}} & \cdots & F_{e_{i}}\overset{}{\cdots }F_{e_{r}} & \cdots & F_{e_{m}} \end{array}]\cdot {\left[\begin{array}{cccccccc} {\ddot{h}}_{l,e_{1}} & {\ddot{h}}_{l,e_{2}} & \cdots & {\ddot{h}}_{l,e_{i}} & \cdots & {\ddot{h}}_{l,e_{r}} & \cdots & {\ddot{h}}_{l,e_{m}} \end{array}\right]}^{T} \end{array}$$

where the subscript l denotes the sensor’s location; the subscript $e_{i}$ and $e_{r}$ denote the $i^{th}$, $r^{th}$ excitation, respectively; and m is the total number of excitations. Equation (19) can be rewritten as:

(20) $${\ddot{x}}_{l}=F_{e}\cdot {\ddot{h}}_{l,e}$$

where $F_{e}=\left[\begin{array}{cccccc} F_{e_{1}} & F_{e_{2}} & \cdots & F_{e_{i}}\overset{}{\cdots }F_{e_{r}} & \cdots & F_{e_{m}} \end{array}\right]$ and ${\ddot{h}}_{l,e}={\left[\begin{array}{cccccccc} {\ddot{h}}_{l,e_{1}} & {\ddot{h}}_{l,e_{2}} & \cdots & {\ddot{h}}_{l,e_{i}} & \cdots & {\ddot{h}}_{l,e_{r}} & \cdots & {\ddot{h}}_{l,e_{m}} \end{array}\right]}^{T}$.

The acceleration response ${\ddot{x}}_{l}$ can also be expressed implicitly in terms of the IRFs according to Equation (9) as:

(21) $$\begin{array}{c} {\ddot{x}}_{l}=H_{l,e_{1}}\cdot f_{e_{1}}+H_{l,e_{2}}\cdot f_{e_{2}}+\cdots +H_{l,e_{i}}\cdot f_{e_{i}}+\cdots +H_{l,e_{r}}\cdot f_{e_{r}}\cdots +H_{l,e_{m}}\cdot f_{e_{m}}\\ \;=\left[\begin{array}{cccccc} H_{l,e_{1}} & H_{l,e_{2}} & \cdots & H_{l,e_{i}}\overset{}{\cdots }H_{l,e_{r}} & \cdots & H_{l,e_{m}} \end{array}\right]\cdot {\left[\begin{array}{cccccccc} f_{e_{1}} & f_{e_{2}} & \cdots & f_{e_{i}} & \cdots & f_{e_{r}} & \cdots & f_{e_{m}} \end{array}\right]}^{T}\\ \;=H_{l,e}f_{e} \end{array}$$

where $H_{l,e}=\left[\begin{array}{cccccc} H_{l,e_{1}} & H_{l,e_{2}} & \cdots & H_{l,e_{i}}\overset{}{\cdots }H_{l,e_{r}} & \cdots & H_{l,e_{m}} \end{array}\right]$ and $f_{e}={\left[\begin{array}{cccccccc} f_{e_{1}} & f_{e_{2}} & \cdots & f_{e_{i}} & \cdots & f_{e_{r}} & \cdots & f_{e_{m}} \end{array}\right]}^{T}$.

#### 2.3.1. The First Transformation Matrix

Law and Lin [41] proposed a dimensionality reduction transformation matrix, in which a coefficient transformation matrix $Q_{i}$ between two sets of IRFs can be expressed as:

(22) $$H_{l,e_{r}}\cdot Q_{i}=H_{l,e_{i}}$$

The transformation matrix $Q_{i}$ can be computed easily with a pseudoinverse from:

(23) $$Q_{i}=H_{l,e_{r}}^{{}^{†}}\cdot H_{l,e_{i}}$$

If the set of IRF ${\ddot{h}}_{l,e_{r}}$ from the $r^{th}$ excitation is taken as the reference set, other sets of IRF ${\ddot{h}}_{l,e_{i}}$ can be transformed using Equation (23), and Equation (21) becomes:

(24) $$\begin{array}{c} {\ddot{x}}_{l}=H_{l,e_{r}}\cdot Q_{1}\cdot f_{e_{1}}+H_{l,e_{r}}\cdot Q_{2}\cdot f_{e_{2}}+\cdots +H_{l,e_{r}}\cdot Q_{i}\cdot f_{e_{i}}+\cdots +H_{l,e_{r}}\cdot f_{e_{r}}+\cdots +H_{l,e_{r}}\cdot Q_{m}\cdot f_{e_{m}}\\ \;=H_{l,e_{r}}\cdot (Q_{1}\cdot f_{e_{1}}+Q_{2}\cdot f_{e_{2}}+\cdots +Q_{i}\cdot f_{e_{i}}+\cdots +f_{e_{r}}+\cdots +Q_{m}\cdot f_{e_{m}})\\ \;=H_{l,e_{r}}\cdot {\hat{f}}_{e} \end{array}$$

where ${\hat{f}}_{e}=(Q_{1}\cdot f_{e_{1}}+Q_{2}\cdot f_{e_{2}}+\cdots +Q_{i}\cdot f_{e_{i}}+\cdots +f_{e_{r}}+\cdots +Q_{m}\cdot f_{e_{m}})$ is the generalized force vector corresponding to the $r^{th}$ excitation. Note that Equation (24) for the structure under multiple excitations is in a form similar to Equation (9) for the structure under single excitation, and the matrices are defined similarly to those in Equation (10). 

We can rewrite Equation (24) similar to Equation (5) using the explicit expression of ${\ddot{h}}_{l,e_{r}}$ as:

(25) $${\ddot{x}}_{l}={\hat{F}}_{e}\cdot {\ddot{h}}_{l,e_{r}}$$

with

(26) $${\hat{F}}_{e}={\left[\begin{array}{cccc} {\hat{f}}_{e}(0) & 0 & \cdots & 0\\ {\hat{f}}_{e}(1) & {\hat{f}}_{e}(0) & \cdots & 0\\ \vdots & \vdots & \ddots & \vdots \\ {\hat{f}}_{e}(n-1) & {\hat{f}}_{e}(n-2) & \cdots & {\hat{f}}_{e}(0) \end{array}\right]}_{n\times n}$$

#### 2.3.2. The Second Transformation Matrix

The relationship between two sets of unit impulse responses is not unique, and the above is just one example. Another example for such a relationship can be found easily as:

(27) $${\ddot{h}}_{l,e_{i}}=Q_{i}{}^{\prime }\cdot {\ddot{h}}_{l,e_{r}}$$

where the transformation matrix $Q_{i}{}^{\prime }$ is a diagonal matrix with the diagonal elements obtained by the division operation between ${\ddot{h}}_{l,e_{i}}$ and ${\ddot{h}}_{l,e_{r}}$ term by term instead of matrix operation.

Thus, Equation (19) can be expressed as:

(28) $$\begin{array}{c} {\ddot{x}}_{l}=F_{e_{1}}\cdot Q_{1}{}^{\prime }\cdot {\ddot{h}}_{l,e_{r}}+F_{e_{2}}\cdot Q_{2}{}^{\prime }\cdot {\ddot{h}}_{l,e_{r}}+\cdots +F_{e_{i}}\cdot Q_{i}{}^{\prime }\cdot {\ddot{h}}_{l,e_{r}}+\cdots +F_{e_{j}}\cdot {\ddot{h}}_{l,e_{r}}+\cdots +F_{e_{m}}\cdot Q_{m}{}^{\prime }\cdot {\ddot{h}}_{l,e_{r}}\\ \;=(F_{e_{1}}\cdot Q_{1}{}^{\prime }+F_{e_{2}}\cdot Q_{2}{}^{\prime }+\cdots +F_{e_{i}}\cdot Q_{i}{}^{\prime }+\cdots +F_{e_{r}}+\cdots F_{e_{m}}\cdot Q_{m}{}^{\prime })\cdot {\ddot{h}}_{l,e_{r}}\\ \;={\hat{F}}_{e}^{\prime }\cdot {\ddot{h}}_{l,e_{r}} \end{array}$$

with ${{\hat{F}}^{\prime }}_{e}=F_{e_{1}}\cdot Q_{1}{}^{\prime }+F_{e_{2}}\cdot Q_{2}{}^{\prime }+\cdots +F_{e_{i}}\cdot Q_{i}{}^{\prime }+\cdots +F_{e_{r}}+\cdots F_{e_{m}}\cdot Q_{m}{}^{\prime }$

It is noted that Equation (28) for the structure under multiple excitations is in the form of Equation (5) under an equivalent excitation, and the matrices are defined similarly to those in Equation (5). Equations (24) and (28) refer to the case of the structure with an equivalent single excitation after transformation of the effects from other excitations to that of the reference excitation. Thereafter, the number of IRFs has been significantly reduced, and they can be solved by wavelet-based method with Equations (13) and (14) or the regularization-based method with Equation (18).

Transformation matrices $Q_{i}^{}$ and $Q_{i}^{\prime }$ are special forms of the relationship between any two sets of IRF functions in the forms of $H_{l,e_{r}}$ and ${\ddot{h}}_{l,e_{r}}$, respectively. The performances of matrix $Q_{i}^{}$ are studied in the following examples, while a comparison of the performances of matrices $Q_{i}^{}$ and $Q_{i}^{\prime }$ will be given in Section 3.2. 

### 2.4. IRF Estimation-Based Optimal Sensor Placement Method

The sensor locations were arbitrarily selected by experience in [41], and it is noted that different sensor placement configuration would give different accuracy in the IRF estimation as well as damage detection result. However, there is no existing optimal sensor placement method suitable for the IRF estimation-based damage detection under multiple seismic excitations. The transformation matrix and the estimation method for IRF extraction may induce some uncertainties for damage detection. To enhance the accuracy of damage detection, an optimal sensor placement method based on the principle of minimizing the estimation error is proposed in this paper. The normalized IRF estimation error β can be written as:

(29) $$\beta =\left\|\frac{{\ddot{h}}_{r}^{0}-{\ddot{h}}_{a}^{0}}{\mathrm{std}({\ddot{h}}_{a}^{0})}\right\|$$

where ${\ddot{h}}_{r}^{0}$ and ${\ddot{h}}_{a}^{0}$ are the estimated IRF obtained from the measured acceleration and the analytical IRF from the finite element model in the initial intact state, and std(•) is the standard deviation operation.

The objective function for selecting the sensor locations with minimum error is expressed as

(30) $$\arg\underset{\theta }{\min}f_{obj}(\theta )=\mathrm{tr}\left[\beta \beta^{T}\right]=\mathrm{diag}\left[\beta \beta^{T}\right]$$

where $f_{obj}(\theta )$ is optimal sensor placement objective function based on the minimization of IRF estimation error β. Thus, the optimal sensor configuration with a prescribed number of sensors can be obtained by solving Equation (30).

### 2.5. IRF Estimation-Based Damage Detection

A sensitivity-based model updating method is adopted for the damage identification. The local damage is assumed only related to a stiffness reduction as $\alpha_{i}K_{i}$, where $K_{i}$ and $\alpha_{i}$ are the elemental stiffness matrix in the intact state and the stiffness reduction factor of the $i^{th}$ element with $0.0\leq \alpha_{i}\leq 1.0$. 

#### 2.5.1. Damage Detection Algorithm

**Figure 2:**
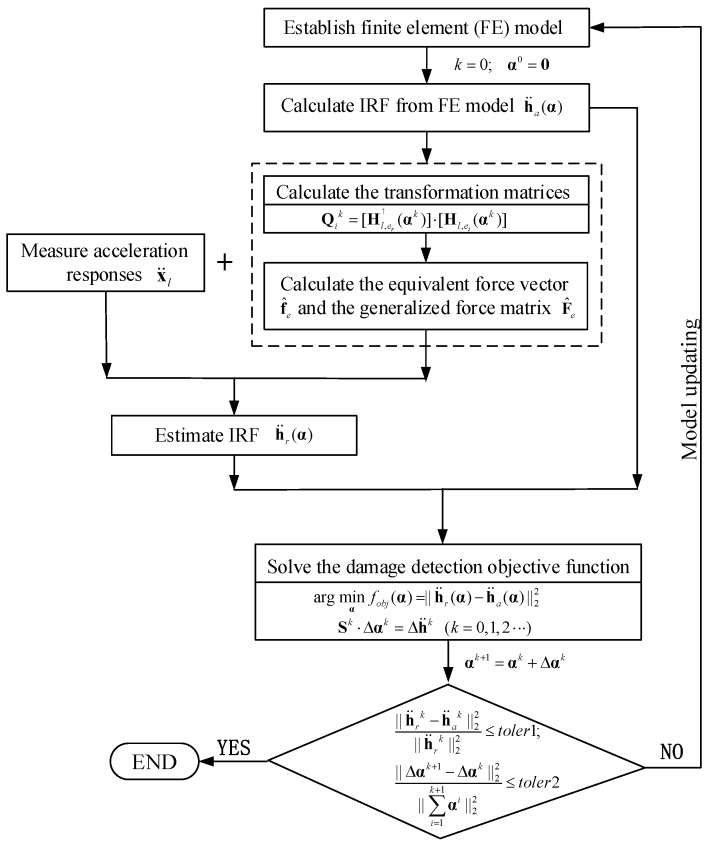
The flowchart of the damage detection by using estimated impulse response function (IRF).

The objective function for damage detection is defined as the difference between two sets of IRF vectors as:

(31) $$\arg\;\underset{\alpha }{\min}f_{obj}(\alpha )=||{\ddot{h}}^{}{}_{r}(\alpha )-{\ddot{h}}^{}{}_{a}(\alpha )|{|}_{2}^{2}$$

where ${\ddot{h}}^{}{}_{r}$ and ${\ddot{h}}^{}{}_{a}$ are the estimated IRF obtained from the measured acceleration in the damage state and the analytical IRF from the finite element model in the initial intact state, respectively. The objective function in Equation (31) is minimized and the vector $\alpha_{i}$ of elemental stiffness factors and the transformation matrices $Q_{i}^{}$ and $Q_{i}^{\prime }$ in the initial intact finite element model are iteratively updated as shown in Figure 2 such that the estimated IRF matches well with the analytical IRF in the damaged state.

The objection function in Equation (31) can be expressed in first-order Taylor expansion as:

(32) $$S\Delta \alpha =\Delta \ddot{h}={\ddot{h}}^{}{}_{r}-{\ddot{h}}^{0}{}_{a}$$

where the sensitivity matrix S is computed with the finite difference method from the analytical model as:

(33) $$S=\left[\frac{{\ddot{h}}_{a}(\alpha +\Delta \alpha )-{\ddot{h}}_{a}(\alpha )}{\Delta \alpha }\right]=\left[\frac{\partial {\ddot{h}}_{a}}{\partial \alpha_{1}},\frac{\partial {\ddot{h}}_{a}}{\partial \alpha_{2}}\cdots \frac{\partial {\ddot{h}}_{a}}{\partial \alpha_{ne}}\right]$$

#### 2.5.2. Model Updating Procedure

The damage detection equation in Equation (32) is solved by using the iterative Gaussian–Newton method with:

(34) $$S^{k}\cdot \Delta \alpha^{k}=\Delta {\ddot{h}}^{k}\overset{}{}(k=0,1,2\cdot \cdot \cdot )$$

where superscript $k,\;\Delta \alpha^{k}$ denote the iteration number and the fractional stiffness reduction at the $k^{\mathrm{th}}$ iteration, respectively. The transformation matrix $Q_{i}^{}$ in Equation (23) takes up the following form initially from the intact state in the iterative calculation:

(35) $$Q_{i}{}^{0}=[H_{l,e_{r}}^{{}^{†}}(\alpha^{0})]\cdot [H_{l,e_{i}}(\alpha^{0})]$$

The stiffness parameter, sensitivity matrix, and the residual vector from each iteration can be further expressed as $\alpha^{k+1}=\alpha^{k}+\Delta \alpha^{k}$, $S^{k}=S^{k}(\alpha^{k})$ and $\Delta {\ddot{h}}^{k}={\left[{\ddot{h}}_{r}(\alpha^{k})\right]}^{k}-{\left[{\ddot{h}}_{a}(\alpha^{k})\right]}^{k}$, respectively, with an initial set of $\alpha^{0}$. A detailed implementation procedure of the proposed algorithm is given below:

**Step 1:**  The analytical IRF ${\ddot{h}}_{a}$ is computed from the analytical finite element model of the structure under multiple unit excitations using Newmark method.
**Step 2:**  The “measured” acceleration responses ${\ddot{x}}_{l}$ acquired from selected points of the structure are computed from the equation of motion of the structure with the analytical finite element model with local damages and under multiple excitations.
**Step 3:**  Select a reference IRF and compute the transformation matrices $Q_{i}^{}$ by Equation (23) in the initial intact state. Then, the equivalent generalized force vector ${\hat{f}}_{e}$ in Equation (10) can be obtained by using Equation (24), and the equivalent generalized force matrix ${\hat{F}}_{e}$ can also be obtained from its definition in Equation (26).
**Step 4:**  The estimated reference IRF ${\ddot{h}}_{l,e_{r}}$ can be obtained by solving Equation (25) with the explicit expression of an IRF shown in Equation (5).
**Step 5:**  Compute the sensitivity matrix S starting from the initial intact analytical model from Equation (32).
**Step 6:**  Obtain the fractional stiffness reduction $\Delta \alpha^{k}$ from Equation (34) with the adaptive Tikhonov regularization technique [43].
**Step 7:**  The analytical model of the structure is updated from $\alpha^{k+1}=\alpha^{k}+\Delta \alpha^{k}$. The sensitivity matrix is updated from $S^{k}=S^{k}(\alpha^{k})$, and the transformation matrix is improved as $Q_{i}{}^{k}=[H_{l,e_{r}}^{{}^{†}}(\alpha^{k})]\cdot [H_{l,e_{i}}(\alpha^{k})]$ for the next iteration.
**Step 8:**  Repeat Steps 1 to 7 until the following convergence criteria are achieved.
  (36) $$\frac{||{\ddot{h}}_{r}{}^{k}-{\ddot{h}}_{a}{}^{k}|{|}_{2}^{2}}{||{\ddot{h}}_{r}{}^{k}|{|}_{2}^{2}}\leq \mathrm{toler}1;\begin{array}{ccc} & & \frac{||\Delta \alpha^{k+1}-\Delta \alpha^{k}|{|}_{2}^{2}}{||\sum_{i=1}^{k+1}\alpha^{i}|{|}_{2}^{2}}\leq \mathrm{toler}2 \end{array}$$

The tolerance limits for convergence criteria toler1 and toler2 have been set equal to $10^{-3}$ and $10^{-5}$, respectively. The maximum iteration number is set equal to 300 in case when the solution is difficult to converge.

## 3. Numerical Studies

### 3.1. The Structure

**Figure 3:**
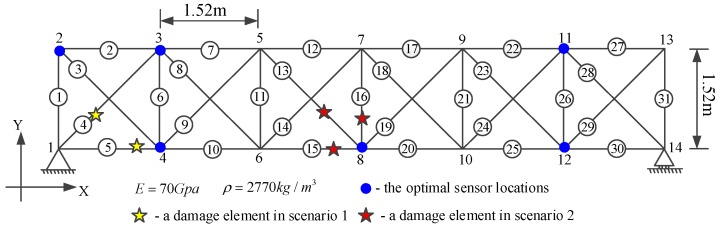
The finite element model of a planar truss structure.

A simply supported plane truss structure served for the simulation study in this paper consists of 31 aluminum alloy bars (1.52 m length, 0.0025 m^2^ cross-sectional area) and 14 nodes (Figure 3). The finite element model of the truss structure is established by using commercial software MATLAB. Each bar of the truss structure is simulated as one finite element. The Young modulus and density of the bar are 70 GPa and ${2770\;\mathrm{kg}/m}^{3}$, respectively. The structure is supported at nodes 1 and 14. Both the vertical and horizontal translational restraints at the supports are represented by a large stiffness of $1.0\times 10^{10}\;\mathrm{kN}/m$. Rayleigh damping is adopted for the system with the first two damping ratios $\xi_{1}=0.01$ and $\xi_{2}=0.005$. The first five natural frequencies are 36.09, 75.63, 132.95, 220.95, and 248.58 Hz, respectively.

**Figure 4:**
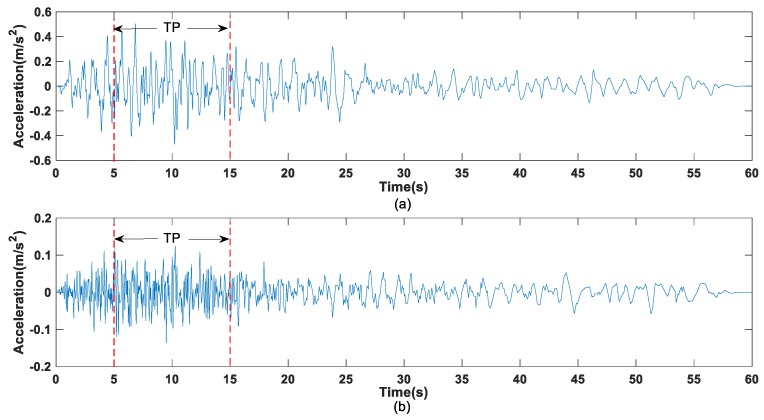
El-Centro seismic acceleration records: (**a**) acting along the horizontal direction (x-axis); (**b**) acting along the vertical direction (y-axis).

Taking advantage of the microtremor of the earth, sufficient energy for damage detection can be provided. The Taiwan Chi-Chi seismic excitation was adopted for a damage detection study based on IRF estimation in [41]. To validate the feasibility of the proposed IRF estimation-based damage detection method under different seismic excitations, the E1-Centro seismic excitations are selected in this study. The 2D truss is subjected to E1-Centro seismic excitations acting along both the x-axis and y-axis of the structure at the supporting nodes without any phase difference. Figure 4a,b show the seismic acceleration records. Generally, excitations with sufficient energy are more effective to detect small damage. Therefore, we selected a time duration with a large excitation amplitude from the seismic records for the study. A ten-second record starting from the 5th to the 15th second are selected from the El-Centro seismic excitation, and it is labeled as TP in Figure 4. The noise effect in the measured acceleration responses is reduced by dividing the responses into 10 equal short data segments for IRF estimation, as shown in Section 3.3. Each data segment lasts for one second and they are named as TP-#1 to TP-#10, respectively, matching the true time histories, where TP-#1 denotes data in the 5th second, TP-#2 represents data in the 6th second, and so on, until TP-#10 stands for data in the 15th second. Only the acceleration response from TP-#1 is used for demonstration of the IRF estimation, optimal sensor placement, and damage detection in the following sections. It is noted in [41] that more data segments can be selected for damage detection by conducting a stability check as some of the data segments may lead to divergence of IRF estimation. To cover the first three modes of the truss, the sampling rate is selected as 300 Hz. Correspondingly, the original seismic acceleration, sampled at 100 Hz, is resampled at 300 Hz through linear interpolation in this study. 

### 3.2. Comparison of Transformation Matrixes $Q_{i}^{}$ and $Q_{i}^{\prime }$

In the derivation of the transformation matrices $Q_{i}^{}$ and $Q_{i}^{\prime }$ above, it is noted that the relationship between two sets of unit impulse responses is not unique and matrices $Q_{i}^{}$ or $Q_{i}^{\prime }$ are just one special form. A comparison of the robustness of the two transformation matrices is needed. It is noted that two different equivalent excitation force matrices ${\hat{f}}_{e}$ and ${\hat{F}}_{e}{}^{\prime }$ with the definitions given in Equations (24) and (28), respectively, can be obtained via the transformation matrices $Q_{i}^{}$ and $Q_{i}^{\prime }$, respectively. A check on the components of equivalent excitation force matrices ${\hat{f}}_{e}$ and ${\hat{F}}_{e}{}^{\prime }$ may serve as a qualitative evaluation of the stability of the transformation matrices indirectly.

**Figure 5:**
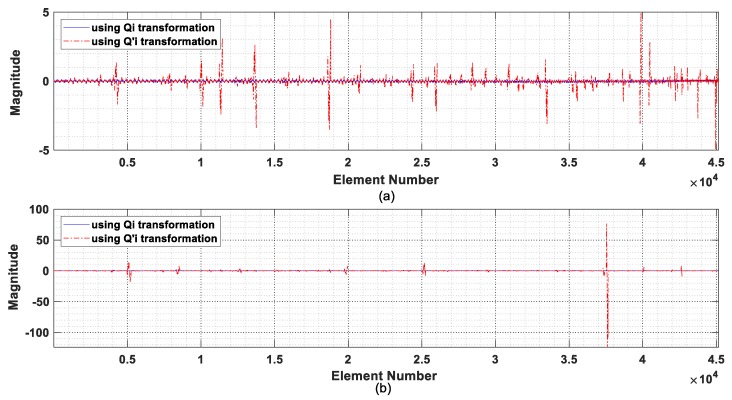
Comparison of the nonzero elements of the generalized excitation force matrix: (**a**) using IRF at node 5; (**b**) using IRF at node 12.

The equivalent excitation force matrices ${\hat{F}}_{e}$ in Equation (26) and ${\hat{F}}_{e}{}^{\prime }$ in Equation (28), respectively, are computed via transformation matrices $Q_{i}^{}$ and $Q_{i}^{\prime }$ for the IRF estimation at nodes 5 and 12 from the intact state with one-second measured acceleration response in the recording denoted as TP-#1. The nonzero elements of equivalent excitation force matrices ${\hat{F}}_{e}$ and ${\hat{F}}_{e}{}^{\prime }$ are plotted and compared in Figure 5. The equivalent excitation force computed from matrix $Q_{i}^{}$ as Equation (6) is noted more stable than that computed from the transformation matrix $Q_{i}^{\prime }$. The latter has large fluctuations in the data series, which are due to the term-by-term division operation with a small denominator for obtaining the transformation matrix $Q_{i}^{\prime }$. 

**Figure 6:**
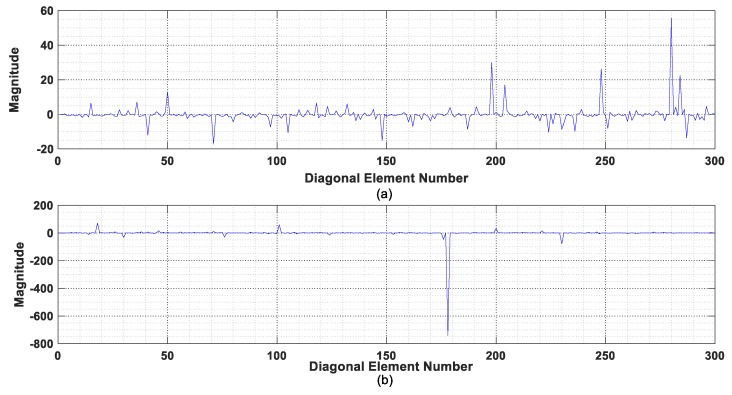
Diagonal elements of matrix $Q_{i}^{\prime }$ for IRF estimation: (**a**) using IRF at node 5; (**b**) using IRF at node 12.

The diagonal elements of matrix $Q_{i}^{\prime }$ obtained from the vertical IRFs at nodes 5 and 12 are plotted in Figure 6. There are large values in the diagonal elements numbered 198, 204, 248, 280, and 284 as shown in Figure 6a, which correspond to the large fluctuations in the nonzero equivalent excitation force in the IRF estimation at node 5 as shown in Figure 5a. Similar observations are found in Figure 5b and Figure 6b for the IRF estimation at node 12, where a distinct large value exists in the diagonal element 178 of $Q_{i}^{\prime }$. It is clearly noted that a large diagonal element of matrix $Q_{i}^{\prime }$ would lead to a large fluctuation in the computed equivalent excitation force. It may therefore be concluded that the transformation matrix $Q_{i}^{}$ has better performance than matrix $Q_{i}^{\prime }$ in the computation, and it will be used in the subsequent studies of IRF estimation and damage detection.

### 3.3. Comparison of Two Methods for IRF Estimation

The estimation of the IRF is, in essence, an inverse problem including the solution of the ill-conditioned equations subject to the effect of measurement noise. The accurate estimation of the IRF is thus one of the obstacles for its application to identify local structural damages. In this section, the wavelet-based and regularization-based methods are compared in the estimation of the IRF in noise contaminated environment. 

**Figure 7:**
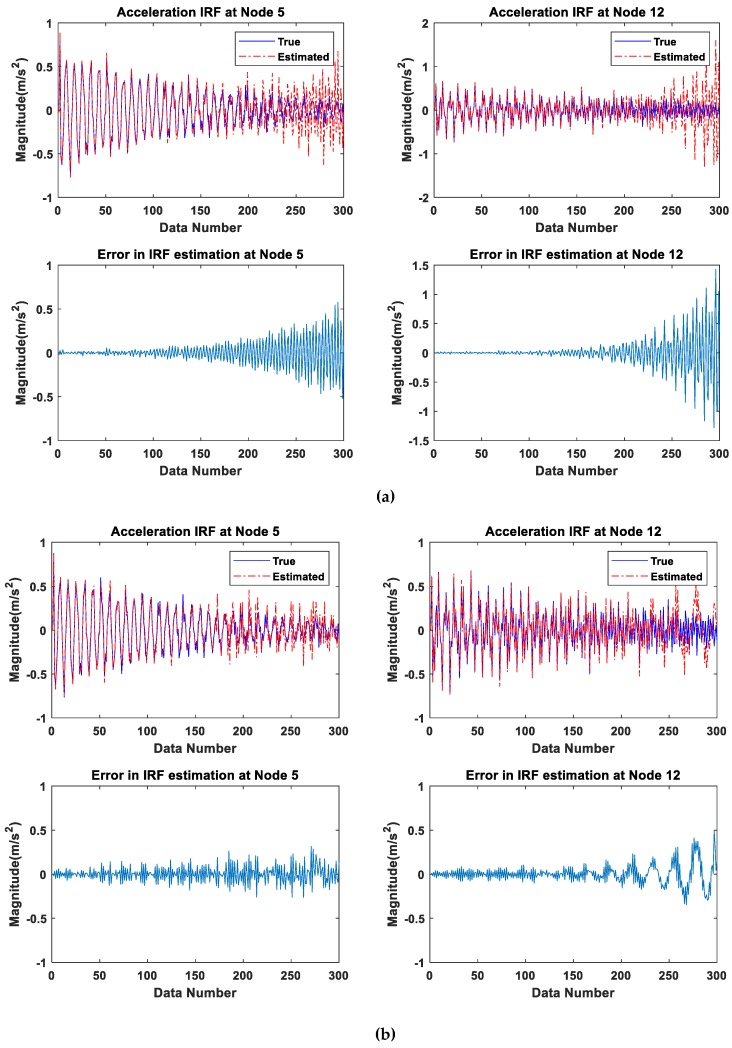
The comparison of the estimated IRFs using different methods: (**a**) Using discrete wavelet transform and least-squares method; (**b**) using the Tikhonov regularization method.

The intact structure is subjected to El-Centro seismic excitation (see Figure 4) and the acceleration responses at nodes 5 and 12 in TP-#1 recording with 5% random noise is used for the estimation of the IRFs. The estimated IRFs via wavelet-based method using Equations (12) and (14) is shown in Figure 7a from 1 × 300 data. The estimated IRF data points ranging from 0–200 match well with the true IRF, while large deviations from the true analytical IRF are found close to the end of the estimated IRF. The estimated IRFs via the regularization-based approach using Equation (18) are demonstrated in Figure 7b. The regularization-based approach is noted to yield estimates on the IRF with higher accuracy. A close inspection in Figure 7 shows that the estimation error increases gradually with time and is particularly noted in the data points towards the end ranging from 200–300. This may be due to the diminishing signal-to-noise ratio with time as the IRF is a decaying function when the response diminishes to zero. According to this property of the IRF, a period of stable estimated IRF can then be selected to reduce the noise effect for subsequent damage detection. 

The regularization-based method may also be concluded to be more effective than the wavelet-based method in estimating the IRF. It is also suggested to have only the first 70% of the estimated IRF from one measurement in one second, i.e., 1 × 300 × 70% = 210 data points for the subsequent damage detection when the sampling ratio is 300 Hz. 

### 3.4. Sensor Placement

**Figure 8:**
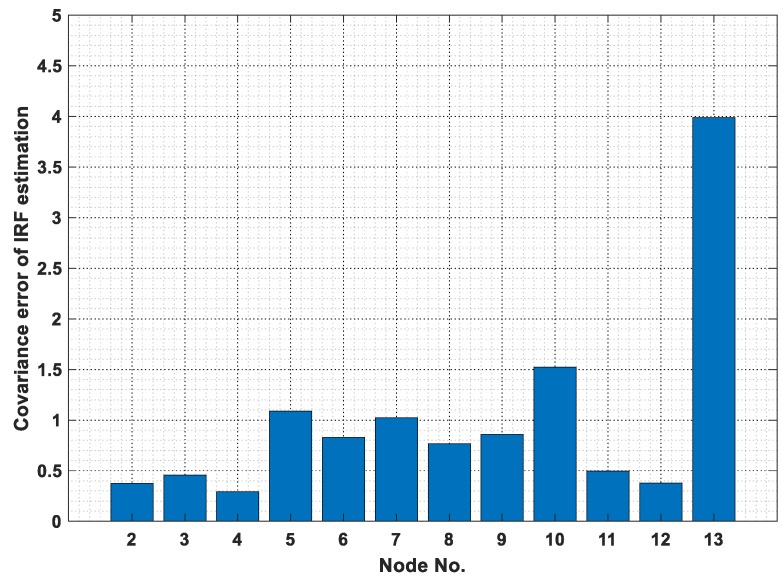
Covariance error of estimation for vertical IRF in the vertical direction.

The structure is assumed to be under both horizontal and vertical El-Centro seismic excitations, as shown in Figure 4. The horizontal IRF at each measured node is transformed taking the vertical IRF at the same node as reference in this study. Twelve accelerometers in the vertical direction from nodes 2 to 13 are selected as candidates for measurement. It is assumed to have only six optimal selecting sensors for damage detection. The optimal sensor configuration is obtained by minimizing Equation (30). The diagonal elements in the matrix $\left[\beta \beta^{T}\right]$ are the covariance error of IRF estimation for the corresponding sensors as shown in Figure 8. Therefore, the sensors installed in the vertical direction of nodes 2, 3, 4, 8, 11, and 12 are selected for the following damage detection studies.

### 3.5. Damage Identification Using the Estimated IRFs

Local damage in the truss structure (Figure 3) is produced by introducing a reduction in the axial stiffness of individual bars and keeping the inertial properties unchanged. Only one damage scenario has been studied in the IRF estimation-based damage detection in [41]. To examine the feasibility and effectiveness of the proposed method for structural damage detection, two multiple damage scenarios are considered and the details are listed in Table 1. In the first scenario, damages are assumed to occur in element 4 (diagonal member) and element 5 (lateral member) close to the support of the structure. Element 4 is of 10% stiffness reduction, while element 5 is of 15% stiffness reduction. In the second scenario, damages occur in a total of three elements at mid-span of the structure: element 13 (diagonal member), element 15 (lateral member), and element 16 (vertical member). Elements 13, 15, and 16 have 15%, 10%, and 15% stiffness reduction, respectively. These unknown damages are detected by solving Equation (32).

**Figure 9:**
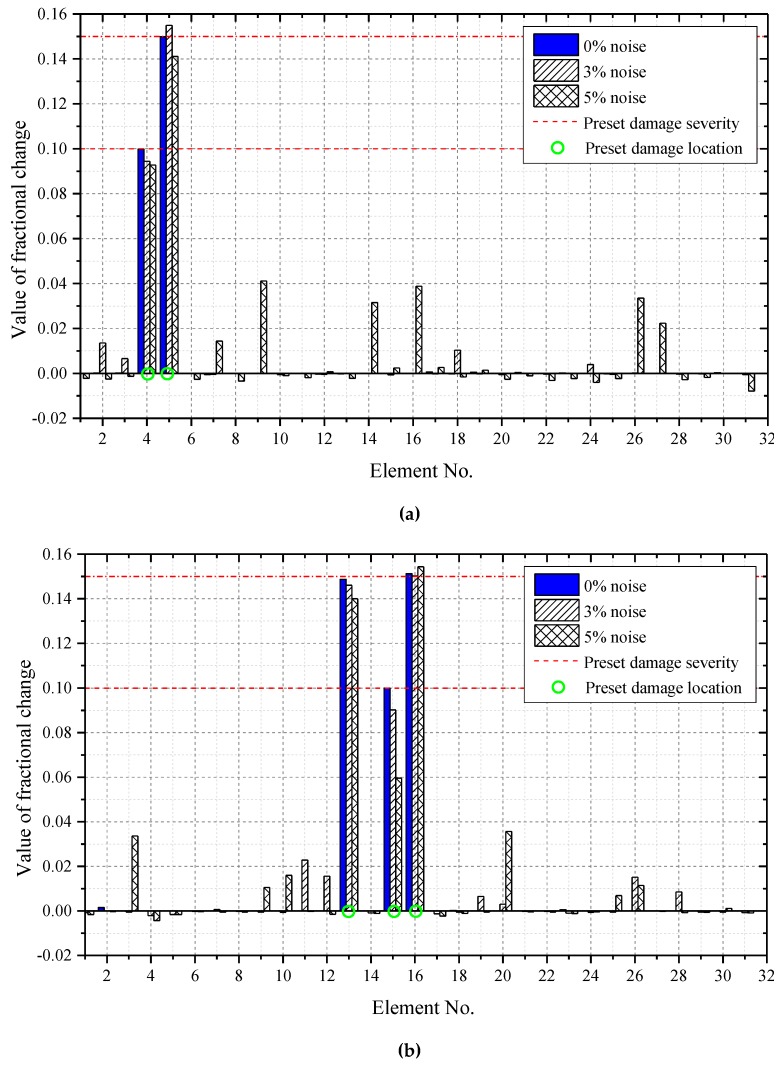
Damage detection results under different noise levels: (**a**) damage scenario 1; (**b**) damage scenario 2.

Acceleration response data from the truss structure under El-Centro seismic excitation as shown in Figure 4 with one-second duration in TP-#1 recording is used for IRF estimation and damage detection study. The damage detection results for the two damage scenarios are depicted in Figure 9a,b. It is noted that both the damage locations and the damage extents can be identified accurately in the noise-free cases. When 3% noise is added to the measured acceleration responses, both the damage locations and the extents can be identified satisfactorily in both damage scenarios. When 5% noise is added, the damage detection accuracy is reduced with false positives. In damage scenario I, the damaged elements 4 and 5 are identified with 9.27% and 14.11% stiffness reduction, respectively. Some large false positives are noted in the undamaged elements 9 (4.11%), 14 (3.16%), 16 (3.88%), 26 (3.35%), and 27 (2.23%). In damage scenario 2, the damaged elements 13, 15, and 16 are identified with 14%, 5.97%, and 15.43% stiffness reduction, respectively. Similarly, large false positives are noted in the undamaged elements 3 (3.37%) and 20 (3.57%). It is believed that the false alarms are generated due to errors in the inverse computation of the IRF estimation and damage detection. It may be concluded that the proposed method for damage detection is feasible and accurate under median-level measurement noise for practical application. In addition, the computation time of the IRF-based damage detection of the truss structure is about 265–280 s for each damage scenario by using the computer with the Intel(R) Core (TM) i7-8550U CPU @1.80 GHz 1.99 GHz and 12.0 G RAM.

## 4. Discussions

The feasibility of structural damage diagnosis based on impulse response function estimation under multiple seismic excitations has been reported above. This approach has the following distinct features:

(a) The dimensionality reduction transformation matrices for IRF estimation is not limited to the seismic excitations. They can be applied to structures subjected to other types of excitations, such as wind loads and traffic loads. The proposed method is potentially applicable to the damage detection of different structures such as bridges, buildings, tunnels, offshore platforms, and ships.
(b) It is important to select an appropriate reference IRF; otherwise, it may lead to a large error in the transformation matrix. This is because of the very small amplitude excited at the inappropriate reference location. To avoid this problem, IRF from different locations can be compared for evaluating their suitability to serve as the reference IRF before conducting the estimation.
(c) Finally, it is noted that the IRF estimation and damage detection are prone to noise contamination. The proposed optimal sensor placement method and the selection of data points in the IRF are effective to alleviate the noise effect. As shown in the numerical study, both the damage locations and the extents can be satisfactorily identified under 3% measurement noise. With the increase of measurement noise to 5%, the damaged elements can be accurately localized, but the precision of identified damage extent is slightly reduced and small false positives occur. It is believed that the reduced identification performance and false positives result from a limited number of sensors and the inverse computation errors of both the IRF estimation and damage detection.

## 5. Conclusions

A methodology for estimating IRF from structural responses resulted from multiple unknown excitations is demonstrated and applied in the damage detection of a structure. Two different transformation matrices are compared for transforming the multiple general excitation problem into an equivalent single excitation problem. The first transformation matrix $Q_{i}^{}$ is selected due to the higher robustness. IRFs are then estimated with the first matrix in two IRF estimation methods, namely the wavelet-based and regularization-based methods. Numerical validation of the proposed estimation method is conducted with a plane truss structure under seismic excitations. The estimation results obtained using the regularization-based method are proved more accurate than the wavelet-based method. Methods to reduce the measurement noise effect on the damage detection results are also proposed, which include a sensor placement method and the selection of IRF data with a large signal-to-noise ratio. It is found that the damage locations and the damage extents are accurately identified in the noise-free cases. Further, the IRF-based damage detection is examined under 3% and 5% measurement noise, and both the damage locations and the damage extents can be identified satisfactorily with only several small false alarms. It can be concluded that the proposed method for damage detection is feasible under median-level measurement noise for practical application.

## Figures and Tables

**Table 1:** Numerical studies scenarios and parameters.

Scenarios	I	II
Damage Element No. Damage Extent (%)	4th (10%) and 5th (15%)	13th (15%), 15th (10%) and 16th (15%)
Sampling Rate (Hz)	300	
Noise Level (%)	0, 3 and 5	

## List of Symbols

C	=	the N×N damping matrix
D	=	the mapping matrix relating the excitation forces to the corresponding DOFs of the structure
$e_{i}$ and $e_{r}$	=	denote the $i^{th}$, $r^{th}$ excitation respectively
f(t)	=	a single excitation
F	=	implicitly expressed matrix form of excitation
$F^{DWT}$	=	$F^{DWT}$ is the force matrix F after discrete wavelet transform
h(t)	=	IRF of displacement vector
$\dot{h}(t)$	=	IRF of velocity vector
$\ddot{h}(t)$	=	IRF of acceleration vector
${\ddot{h}}_{l}{}^{DWT}$	=	the unit impulse response vector ${\ddot{h}}_{l}$ after discrete wavelet transform
${\ddot{h}}_{l}(t)$	=	IRF of acceleration response from location l at time t
${\ddot{h}}^{}{}_{r}$	=	the estimated IRF obtained from the measured acceleration in the damage state
${\ddot{h}}^{}{}_{a}$	=	the analytical IRF from the finite element model in the initial intact state
$H_{l}$	=	implicitly expressed matrix form of ${\ddot{h}}_{l}(t)$
I	=	the identity matrix
K	=	the N×N global stiffness matrix
M	=	the N×N mass matrix
n	=	the total number of time instants
p	=	the number of data points
$Q_{i}$	=	the first dimensionality reduction transformation matrix
$Q_{i}{}^{\prime }$	=	the second dimensionality reduction transformation matrix
S	=	the sensitivity matrix
U	=	the left singular matrix
$u_{1},u_{2},\cdots ,u_{m}$	=	the left singular vectors
V	=	the right singular matrix
$v_{1}^{},v_{2}^{},\cdots ,v_{n}^{}$	=	the right singular vector
x(t)	=	the displacement vector
$\dot{x}(t)$	=	the velocity vector
$\ddot{x}(t)$	=	the acceleration vector
${\ddot{x}}_{l}(t)$	=	the acceleration response from location l at time t
$\alpha_{i}$	=	the stiffness reduction factor of the $i^{th}$ element
Δα	=	the value of factional change of stiffness reduction
β	=	the normalized IRF estimation error
δ(t)	=	the Dirac delta function
λ	=	the regularization parameter
$\xi_{1}$ and $\xi_{2}$	=	the first two damping ratios
$\sigma_{1},\sigma_{2},\cdots ,\sigma_{r}$	=	singular values
Σ	=	the diagonal matrix of singular values
φ(τ)	=	the scaling function
ψ(τ)	=	the mother wavelet function
**List of Abbreviations**		
CoC	=	covariance of covariance
DOFs	=	degrees-of-freedom
FRF	=	frequency response function
DWT	=	discrete wavelet transform
IDWT	=	inverse discrete wavelet transform
IRF	=	impulse response function
SHM	=	structural health monitoring
std(•)	=	standard deviation operation
